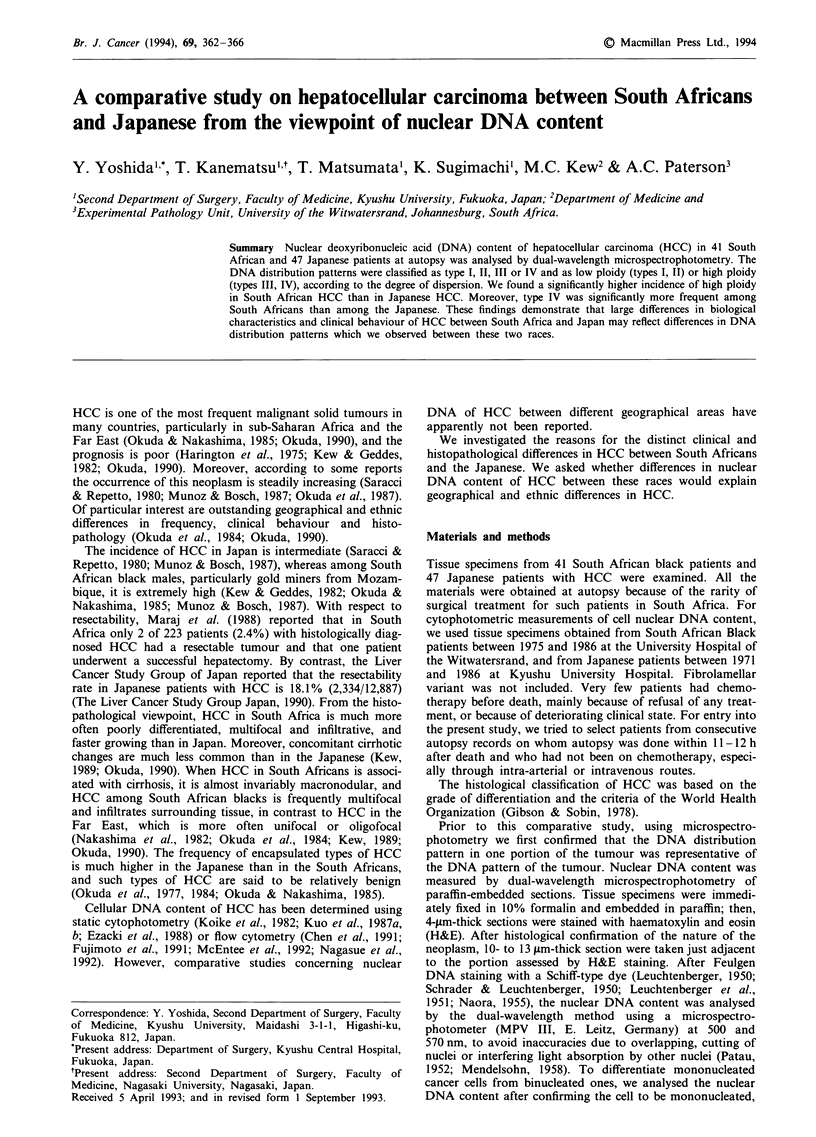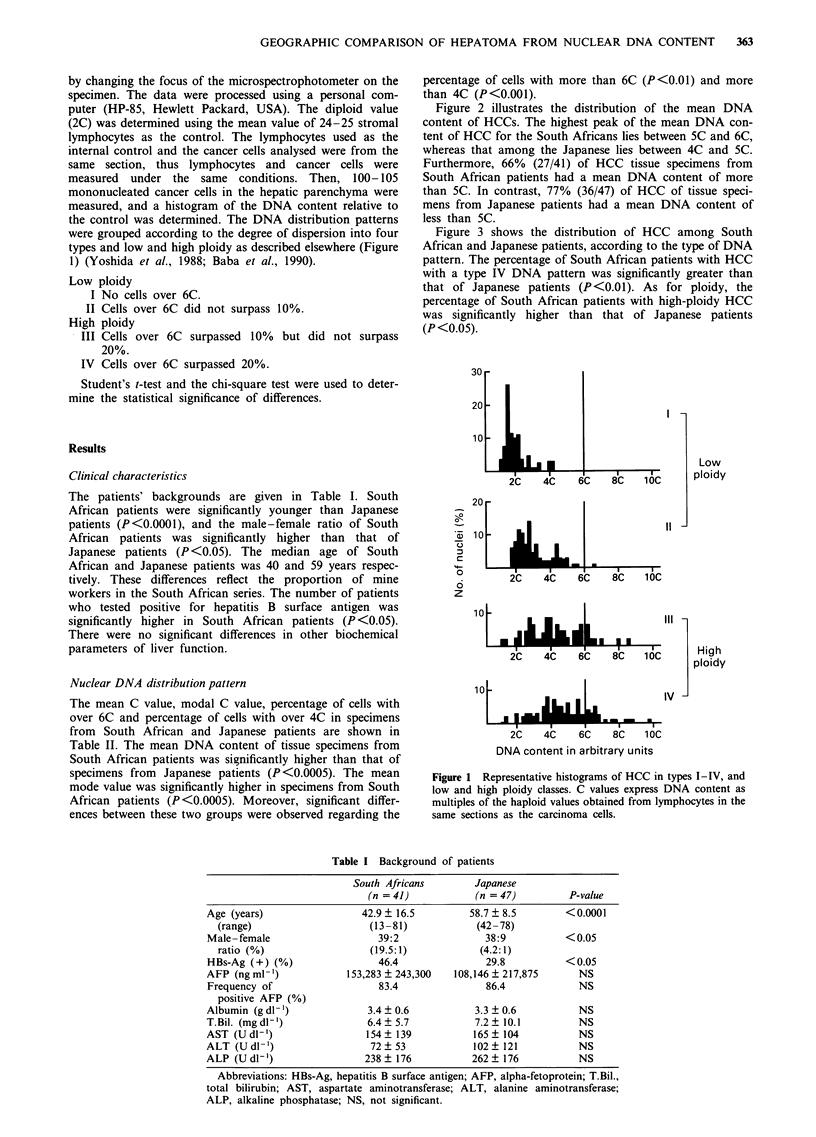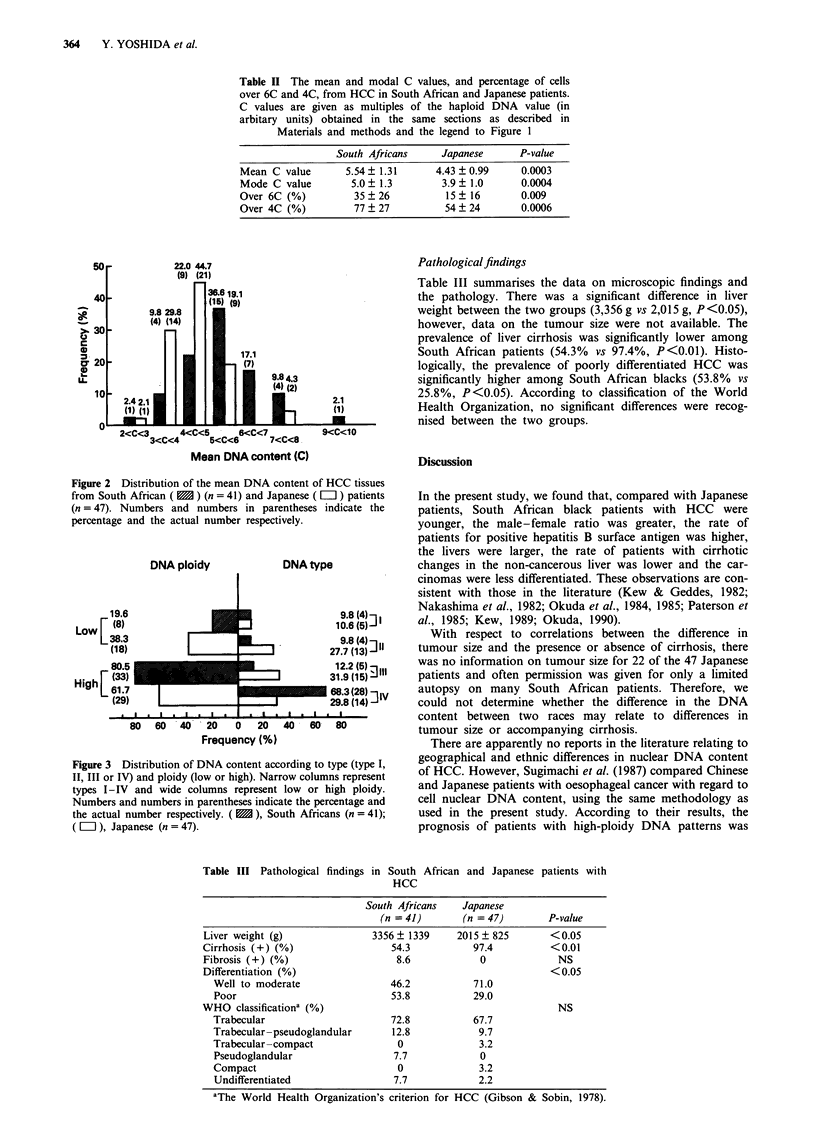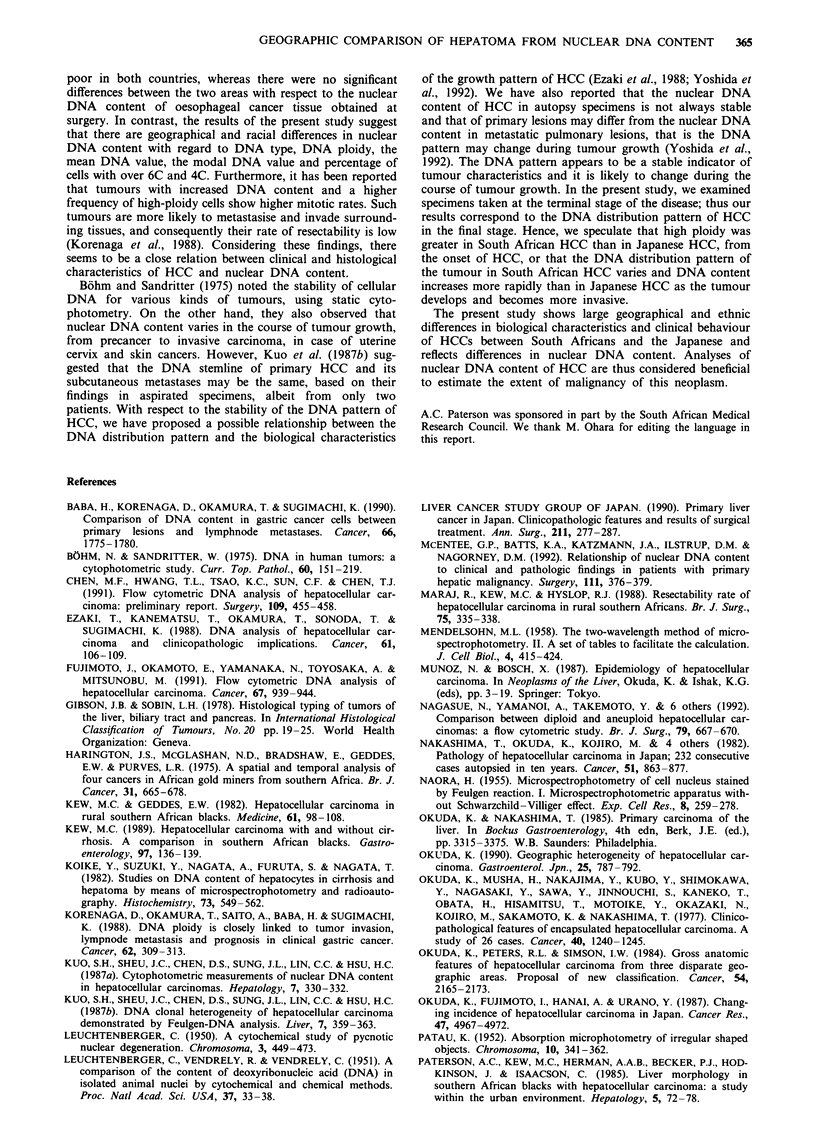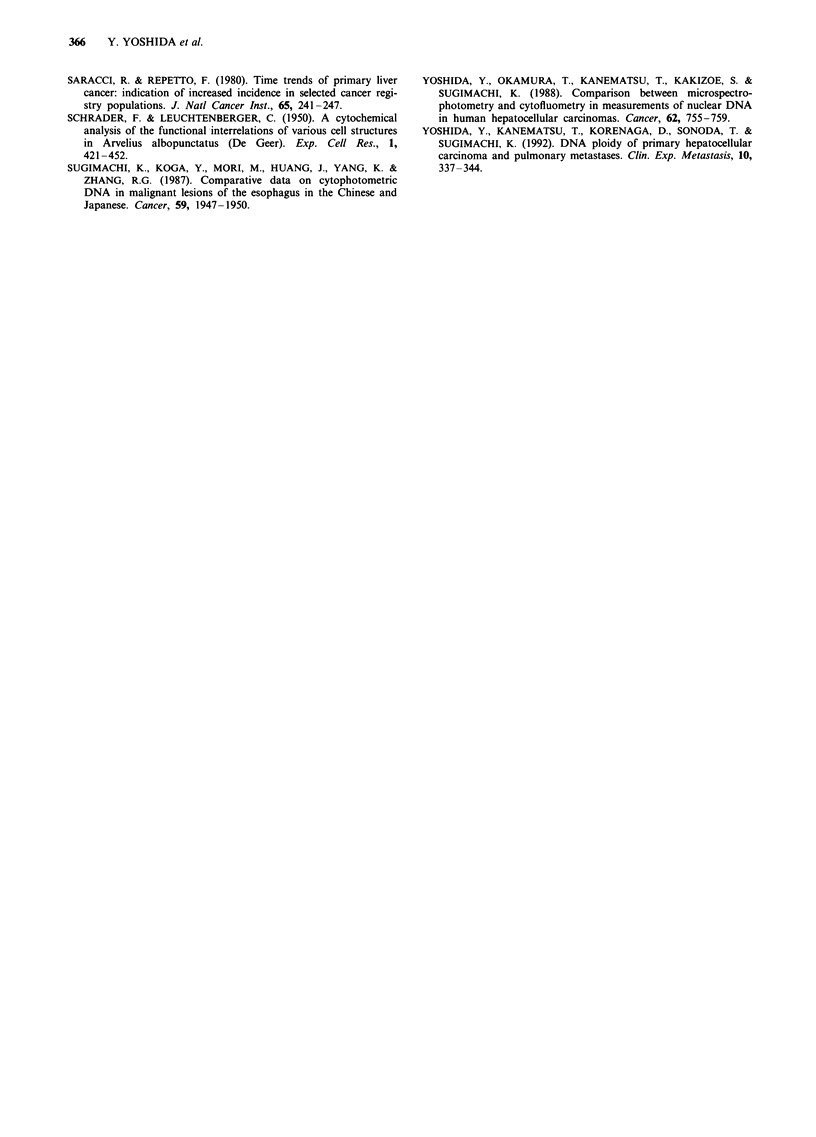# A comparative study on hepatocellular carcinoma between South Africans and Japanese from the viewpoint of nuclear DNA content.

**DOI:** 10.1038/bjc.1994.66

**Published:** 1994-02

**Authors:** Y. Yoshida, T. Kanematsu, T. Matsumata, K. Sugimachi, M. C. Kew, A. C. Paterson

**Affiliations:** Second Department of Surgery, Faculty of Medicine, Kyushu University, Fukuoka, Japan.

## Abstract

Nuclear deoxyribonucleic acid (DNA) content of hepatocellular carcinoma (HCC) in 41 South African and 47 Japanese patients at autopsy was analysed by dual-wavelength microspectrophotometry. The DNA distribution patterns were classified as type I, II, III or IV and as low ploidy (types I, II) or high ploidy (types III, IV), according to the degree of dispersion. We found a significantly higher incidence of high ploidy in South African HCC than in Japanese HCC. Moreover, type IV was significantly more frequent among South Africans than among the Japanese. These findings demonstrate that large differences in biological characteristics and clinical behaviour of HCC between South Africa and Japan may reflect differences in DNA distribution patterns which we observed between these two races.


Br. J. Cancer (1994), 69, 362–366 © Macmillan Press Ltd., 1994

### A comparative study on hepatocellular carcinoma between South Africans and Japanese from the viewpoint of nuclear DNA content

Y. Yoshida[1,*], T. Kanematsu[1,†], T. Matsumata[1], K. Sugimachi[1], M.C. Kew[2] & A.C. Paterson[3]

[1]*Second Department of Surgery, Faculty of Medicine, Kyushu University, Fukuoka, Japan;* [2]*Department of Medicine and* [3]*Experimental Pathology Unit, University of the Witwatersrand, Johannesburg, South Africa.*

**Summary** Nuclear deoxyribonucleic acid (DNA) content of hepatocellular carcinoma (HCC) in 41 South African and 47 Japanese patients at autopsy was analysed by dual-wavelength microspectrophotometry. The DNA distribution patterns were classified as type I, II, III or IV and as low ploidy (types I, II) or high ploidy (types III, IV), according to the degree of dispersion. We found a significantly higher incidence of high ploidy in South African HCC than in Japanese HCC. Moreover, type IV was significantly more frequent among South Africans than among the Japanese. These findings demonstrate that large differences in biological characteristics and clinical behaviour of HCC between South Africa and Japan may reflect differences in DNA distribution patterns which we observed between these two races.

HCC is one of the most frequent malignant solid tumours in many countries, particularly in sub-Saharan Africa and the Far East (Okuda & Nakashima, 1985; Okuda, 1990), and the prognosis is poor (Harington *et al.*, 1975; Kew & Geddes, 1982; Okuda, 1990). Moreover, according to some reports the occurrence of this neoplasm is steadily increasing (Saracci & Repetto, 1980; Munoz & Bosch, 1987; Okuda *et al.*, 1987). Of particular interest are outstanding geographical and ethnic differences in frequency, clinical behaviour and histopathology (Okuda *et al.*, 1984; Okuda, 1990).

The incidence of HCC in Japan is intermediate (Saracci & Repetto, 1980; Munoz & Bosch, 1987), whereas among South African black males, particularly gold miners from Mozambique, it is extremely high (Kew & Geddes, 1982; Okuda & Nakashima, 1985; Munoz & Bosch, 1987). With respect to resectability, Maraj *et al.* (1988) reported that in South Africa only 2 of 223 patients (2.4%) with histologically diagnosed HCC had a resectable tumour and that one patient underwent a successful hepatectomy. By contrast, the Liver Cancer Study Group of Japan reported that the resectability rate in Japanese patients with HCC is 18.1% (2,334/12,887) (The Liver Cancer Study Group Japan, 1990). From the histopathological viewpoint, HCC in South Africa is much more often poorly differentiated, multifocal and infiltrative, and faster growing than in Japan. Moreover, concomitant cirrhotic changes are much less common than in the Japanese (Kew, 1989; Okuda, 1990). When HCC in South Africans is associated with cirrhosis, it is almost invariably macronodular, and HCC among South African blacks is frequently multifocal and infiltrates surrounding tissue, in contrast to HCC in the Far East, which is more often unifocal or oligofocal (Nakashima *et al.*, 1982; Okuda *et al.*, 1984; Kew, 1989; Okuda, 1990). The frequency of encapsulated types of HCC is much higher in the Japanese than in the South Africans, and such types of HCC are said to be relatively benign (Okuda *et al.*, 1977, 1984; Okuda & Nakashima, 1985).

Cellular DNA content of HCC has been determined using static cytophotometry (Koike *et al.*, 1982; Kuo *et al.*, 1987*a*, *b*; Ezacki *et al.*, 1988) or flow cytometry (Chen *et al.*, 1991; Fujimoto *et al.*, 1991; McEntee *et al.*, 1992; Nagasue *et al.*, 1992). However, comparative studies concerning nuclear DNA of HCC between different geographical areas have apparently not been reported.

We investigated the reasons for the distinct clinical and histopathological differences in HCC between South Africans and the Japanese. We asked whether differences in nuclear DNA content of HCC between these races would explain geographical and ethnic differences in HCC.

### Materials and methods

Tissue specimens from 41 South African black patients and 47 Japanese patients with HCC were examined. All the materials were obtained at autopsy because of the rarity of surgical treatment for such patients in South Africa. For cytophotometric measurements of cell nuclear DNA content, we used tissue specimens obtained from South African Black patients between 1975 and 1986 at the University Hospital of the Witwatersrand, and from Japanese patients between 1971 and 1986 at Kyushu University Hospital. Fibrolamellar variant was not included. Very few patients had chemotherapy before death, mainly because of refusal of any treatment, or because of deteriorating clinical state. For entry into the present study, we tried to select patients from consecutive autopsy records on whom autopsy was done within 11–12 h after death and who had not been on chemotherapy, especially through intra-arterial or intravenous routes.

The histological classification of HCC was based on the grade of differentiation and the criteria of the World Health Organization (Gibson & Sobin, 1978).

Prior to this comparative study, using microspectrophotometry we first confirmed that the DNA distribution pattern in one portion of the tumour was representative of the DNA pattern of the tumour. Nuclear DNA content was measured by dual-wavelength microspectrophotometry of paraffin-embedded sections. Tissue specimens were immediately fixed in 10% formalin and embedded in paraffin; then, 4-μm-thick sections were stained with haematoxylin and eosin (H&E). After histological confirmation of the nature of the neoplasm, 10- to 13 μm-thick section were taken just adjacent to the portion assessed by H&E staining. After Feulgen DNA staining with a Schiff-type dye (Leuchtenberger, 1950; Schrader & Leuchtenberger, 1950; Leuchtenberger *et al.*, 1951; Naora, 1955), the nuclear DNA content was analysed by the dual-wavelength method using a microspectrophotometer (MPV III, E. Leitz, Germany) at 500 and 570 nm, to avoid inaccuracies due to overlapping, cutting of nuclei or interfering light absorption by other nuclei (Patau, 1952; Mendelsohn, 1958). To differentiate mononucleated cancer cells from binucleated ones, we analysed the nuclear DNA content after confirming the cell to be mononucleated,

Correspondence: Y. Yoshida, Second Department of Surgery, Faculty of Medicine, Kyushu University, Maidashi 3-1-1, Higashi-ku, Fukuoka 812, Japan.
*Present address: Department of Surgery, Kyushu Central Hospital, Fukuoka, Japan.
†Present address: Second Department of Surgery, Faculty of Medicine, Nagasaki University, Nagasaki, Japan.
Received 5 April 1993; and in revised form 1 September 1993.

by changing the focus of the microspectrophotometer on the specimen. The data were processed using a personal computer (HP-85, Hewlett Packard, USA). The diploid value (2C) was determined using the mean value of 24–25 stromal lymphocytes as the control. The lymphocytes used as the internal control and the cancer cells analysed were from the same section, thus lymphocytes and cancer cells were measured under the same conditions. Then, 100–105 mononucleated cancer cells in the hepatic parenchyma were measured, and a histogram of the DNA content relative to the control was determined. The DNA distribution patterns were grouped according to the degree of dispersion into four types and low and high ploidy as described elsewhere (Figure 1) (Yoshida *et al.*, 1988; Baba *et al.*, 1990).

Low ploidy
- I No cells over 6C.
- II Cells over 6C did not surpass 10%.

High ploidy
- III Cells over 6C surpassed 10% but did not surpass 20%.
- IV Cells over 6C surpassed 20%.

Student's *t*-test and the chi-square test were used to determine the statistical significance of differences.

### Results

### *Clinical characteristics*

The patients' backgrounds are given in Table I. South African patients were significantly younger than Japanese patients ($P<0.0001$), and the male–female ratio of South African patients was significantly higher than that of Japanese patients ($P<0.05$). The median age of South African and Japanese patients was 40 and 59 years respectively. These differences reflect the proportion of mine workers in the South African series. The number of patients who tested positive for hepatitis B surface antigen was significantly higher in South African patients ($P<0.05$). There were no significant differences in other biochemical parameters of liver function.

### *Nuclear DNA distribution pattern*

The mean C value, modal C value, percentage of cells with over 6C and percentage of cells with over 4C in specimens from South African and Japanese patients are shown in Table II. The mean DNA content of tissue specimens from South African patients was significantly higher than that of specimens from Japanese patients ($P<0.0005$). The mean mode value was significantly higher in specimens from South African patients ($P<0.0005$). Moreover, significant differences between these two groups were observed regarding the percentage of cells with more than 6C ($P<0.01$) and more than 4C ($P<0.001$).

Figure 2 illustrates the distribution of the mean DNA content of HCCs. The highest peak of the mean DNA content of HCC for the South Africans lies between 5C and 6C, whereas that among the Japanese lies between 4C and 5C. Furthermore, 66% (27/41) of HCC tissue specimens from South African patients had a mean DNA content of more than 5C. In contrast, 77% (36/47) of HCC of tissue specimens from Japanese patients had a mean DNA content of less than 5C.

Figure 3 shows the distribution of HCC among South African and Japanese patients, according to the type of DNA pattern. The percentage of South African patients with HCC with a type IV DNA pattern was significantly greater than that of Japanese patients ($P<0.01$). As for ploidy, the percentage of South African patients with high-ploidy HCC was significantly higher than that of Japanese patients ($P<0.05$).

**Figure 1** Representative histograms of HCC in types I–IV, and low and high ploidy classes. C values express DNA content as multiples of the haploid values obtained from lymphocytes in the same sections as the carcinoma cells.

**Table I** Background of patients

| | *South Africans (n = 41)* | *Japanese (n = 47)* | *P-value* |
|---|---|---|---|
| Age (years) | 42.9 ± 16.5 | 58.7 ± 8.5 | <0.0001 |
| (range) | (13–81) | (42–78) | |
| Male–female | 39:2 | 38:9 | <0.05 |
| ratio (%) | (19.5:1) | (4.2:1) | |
| HBs-Ag (+) (%) | 46.4 | 29.8 | <0.05 |
| AFP (ng ml$^{-1}$) | 153,283 ± 243,300 | 108,146 ± 217,875 | NS |
| Frequency of positive AFP (%) | 83.4 | 86.4 | NS |
| Albumin (g dl$^{-1}$) | 3.4 ± 0.6 | 3.3 ± 0.6 | NS |
| T.Bil. (mg dl$^{-1}$) | 6.4 ± 5.7 | 7.2 ± 10.1 | NS |
| AST (U dl$^{-1}$) | 154 ± 139 | 165 ± 104 | NS |
| ALT (U dl$^{-1}$) | 72 ± 53 | 102 ± 121 | NS |
| ALP (U dl$^{-1}$) | 238 ± 176 | 262 ± 176 | NS |

Abbreviations: HBs-Ag, hepatitis B surface antigen; AFP, alpha-fetoprotein; T.Bil., total bilirubin; AST, aspartate aminotransferase; ALT, alanine aminotransferase; ALP, alkaline phosphatase; NS, not significant.

**Table II** The mean and modal C values, and percentage of cells over 6C and 4C, from HCC in South African and Japanese patients. C values are given as multiples of the haploid DNA value (in arbitary units) obtained in the same sections as described in Materials and methods and the legend to Figure 1

| | *South Africans* | *Japanese* | *P-value* |
|---|---|---|---|
| Mean C value | 5.54 ± 1.31 | 4.43 ± 0.99 | 0.0003 |
| Mode C value | 5.0 ± 1.3 | 3.9 ± 1.0 | 0.0004 |
| Over 6C (%) | 35 ± 26 | 15 ± 16 | 0.009 |
| Over 4C (%) | 77 ± 27 | 54 ± 24 | 0.0006 |

**Figure 2** Distribution of the mean DNA content of HCC tissues from South African ( ▨ ) ($n = 41$) and Japanese ( ☐ ) patients ($n = 47$). Numbers and numbers in parentheses indicate the percentage and the actual number respectively.

**Figure 3** Distribution of DNA content according to type (type I, II, III or IV) and ploidy (low or high). Narrow columns represent types I–IV and wide columns represent low or high ploidy. Numbers and numbers in parentheses indicate the percentage and the actual number respectively. ( ▨ ), South Africans ($n = 41$); ( ☐ ), Japanese ($n = 47$).

*Pathological findings*

Table III summarises the data on microscopic findings and the pathology. There was a significant difference in liver weight between the two groups (3,356 g *vs* 2,015 g, $P < 0.05$), however, data on the tumour size were not available. The prevalence of liver cirrhosis was significantly lower among South African patients (54.3% *vs* 97.4%, $P < 0.01$). Histologically, the prevalence of poorly differentiated HCC was significantly higher among South African blacks (53.8% *vs* 25.8%, $P < 0.05$). According to classification of the World Health Organization, no significant differences were recognised between the two groups.

### Discussion

In the present study, we found that, compared with Japanese patients, South African black patients with HCC were younger, the male–female ratio was greater, the rate of patients for positive hepatitis B surface antigen was higher, the livers were larger, the rate of patients with cirrhotic changes in the non-cancerous liver was lower and the carcinomas were less differentiated. These observations are consistent with those in the literature (Kew & Geddes, 1982; Nakashima *et al.*, 1982; Okuda *et al.*, 1984, 1985; Paterson *et al.*, 1985; Kew, 1989; Okuda, 1990).

With respect to correlations between the difference in tumour size and the presence or absence of cirrhosis, there was no information on tumour size for 22 of the 47 Japanese patients and often permission was given for only a limited autopsy on many South African patients. Therefore, we could not determine whether the difference in the DNA content between two races may relate to differences in tumour size or accompanying cirrhosis.

There are apparently no reports in the literature relating to geographical and ethnic differences in nuclear DNA content of HCC. However, Sugimachi *et al.* (1987) compared Chinese and Japanese patients with oesophageal cancer with regard to cell nuclear DNA content, using the same methodology as used in the present study. According to their results, the prognosis of patients with high-ploidy DNA patterns was

**Table III** Pathological findings in South African and Japanese patients with HCC

| | *South Africans (n = 41)* | *Japanese (n = 47)* | *P-value* |
|---|---|---|---|
| Liver weight (g) | 3356 ± 1339 | 2015 ± 825 | <0.05 |
| Cirrhosis (+) (%) | 54.3 | 97.4 | <0.01 |
| Fibrosis (+) (%) | 8.6 | 0 | NS |
| Differentiation (%) | | | <0.05 |
| Well to moderate | 46.2 | 71.0 | |
| Poor | 53.8 | 29.0 | |
| WHO classification[a] (%) | | | NS |
| Trabecular | 72.8 | 67.7 | |
| Trabecular–pseudoglandular | 12.8 | 9.7 | |
| Trabecular–compact | 0 | 3.2 | |
| Pseudoglandular | 7.7 | 0 | |
| Compact | 0 | 3.2 | |
| Undifferentiated | 7.7 | 2.2 | |

[a]The World Health Organization's criterion for HCC (Gibson & Sobin, 1978).

poor in both countries, whereas there were no significant differences between the two areas with respect to the nuclear DNA content of oesophageal cancer tissue obtained at surgery. In contrast, the results of the present study suggest that there are geographical and racial differences in nuclear DNA content with regard to DNA type, DNA ploidy, the mean DNA value, the modal DNA value and percentage of cells with over 6C and 4C. Furthermore, it has been reported that tumours with increased DNA content and a higher frequency of high-ploidy cells show higher mitotic rates. Such tumours are more likely to metastasise and invade surrounding tissues, and consequently their rate of resectability is low (Korenaga *et al.*, 1988). Considering these findings, there seems to be a close relation between clinical and histological characteristics of HCC and nuclear DNA content.

Böhm and Sandritter (1975) noted the stability of cellular DNA for various kinds of tumours, using static cytophotometry. On the other hand, they also observed that nuclear DNA content varies in the course of tumour growth, from precancer to invasive carcinoma, in case of uterine cervix and skin cancers. However, Kuo *et al.* (1987*b*) suggested that the DNA stemline of primary HCC and its subcutaneous metastases may be the same, based on their findings in aspirated specimens, albeit from only two patients. With respect to the stability of the DNA pattern of HCC, we have proposed a possible relationship between the DNA distribution pattern and the biological characteristics of the growth pattern of HCC (Ezaki *et al.*, 1988; Yoshida *et al.*, 1992). We have also reported that the nuclear DNA content of HCC in autopsy specimens is not always stable and that of primary lesions may differ from the nuclear DNA content in metastatic pulmonary lesions, that is the DNA pattern may change during tumour growth (Yoshida *et al.*, 1992). The DNA pattern appears to be a stable indicator of tumour characteristics and it is likely to change during the course of tumour growth. In the present study, we examined specimens taken at the terminal stage of the disease; thus our results correspond to the DNA distribution pattern of HCC in the final stage. Hence, we speculate that high ploidy was greater in South African HCC than in Japanese HCC, from the onset of HCC, or that the DNA distribution pattern of the tumour in South African HCC varies and DNA content increases more rapidly than in Japanese HCC as the tumour develops and becomes more invasive.

The present study shows large geographical and ethnic differences in biological characteristics and clinical behaviour of HCCs between South Africans and the Japanese and reflects differences in nuclear DNA content. Analyses of nuclear DNA content of HCC are thus considered beneficial to estimate the extent of malignancy of this neoplasm.

A.C. Paterson was sponsored in part by the South African Medical Research Council. We thank M. Ohara for editing the language in this report.

### References

BABA, H., KORENAGA, D., OKAMURA, T. & SUGIMACHI, K. (1990). Comparison of DNA content in gastric cancer cells between primary lesions and lymphnode metastases. *Cancer*, **66**, 1775–1780.

BÖHM, N. & SANDRITTER, W. (1975). DNA in human tumors: a cytophotometric study. *Curr. Top. Pathol.*, **60**, 151–219.

CHEN, M.F., HWANG, T.L., TSAO, K.C., SUN, C.F. & CHEN, T.J. (1991). Flow cytometric DNA analysis of hepatocellular carcinoma: preliminary report. *Surgery*, **109**, 455–458.

EZAKI, T., KANEMATSU, T., OKAMURA, T., SONODA, T. & SUGIMACHI, K. (1988). DNA analysis of hepatocellular carcinoma and clinicopathologic implications. *Cancer*, **61**, 106–109.

FUJIMOTO, J., OKAMOTO, E., YAMANAKA, N., TOYOSAKA, A. & MITSUNOBU, M. (1991). Flow cytometric DNA analysis of hepatocellular carcinoma. *Cancer*, **67**, 939–944.

GIBSON, J.B. & SOBIN, L.H. (1978). Histological typing of tumors of the liver, biliary tract and pancreas. In *International Histological Classification of Tumours, No. 20* pp. 19–25. World Health Organization: Geneva.

HARINGTON, J.S., MCGLASHAN, N.D., BRADSHAW, E., GEDDES, E.W. & PURVES, L.R. (1975). A spatial and temporal analysis of four cancers in African gold miners from southern Africa. *Br. J. Cancer*, **31**, 665–678.

KEW, M.C. & GEDDES, E.W. (1982). Hepatocellular carcinoma in rural southern African blacks. *Medicine*, **61**, 98–108.

KEW, M.C. (1989). Hepatocellular carcinoma with and without cirrhosis. A comparison in southern African blacks. *Gastroenterology*, **97**, 136–139.

KOIKE, Y., SUZUKI, Y., NAGATA, A., FURUTA, S. & NAGATA, T. (1982). Studies on DNA content of hepatocytes in cirrhosis and hepatoma by means of microspectrophotometry and radioautography. *Histochemistry*, **73**, 549–562.

KORENAGA, D., OKAMURA, T., SAITO, A., BABA, H. & SUGIMACHI, K. (1988). DNA ploidy is closely linked to tumor invasion, lympnode metastasis and prognosis in clinical gastric cancer. *Cancer*, **62**, 309–313.

KUO, S.H., SHEU, J.C., CHEN, D.S., SUNG, J.L., LIN, C.C. & HSU, H.C. (1987*a*). Cytophotometric measurements of nuclear DNA content in hepatocellular carcinomas. *Hepatology*, **7**, 330–332.

KUO, S.H., SHEU, J.C., CHEN, D.S., SUNG, J.L., LIN, C.C. & HSU, H.C. (1987*b*). DNA clonal heterogeneity of hepatocellular carcinoma demonstrated by Feulgen-DNA analysis. *Liver*, **7**, 359–363.

LEUCHTENBERGER, C. (1950). A cytochemical study of pycnotic nuclear degeneration. *Chromosoma*, **3**, 449–473.

LEUCHTENBERGER, C., VENDRELY, R. & VENDRELY, C. (1951). A comparison of the content of deoxyribonucleic acid (DNA) in isolated animal nuclei by cytochemical and chemical methods. *Proc. Natl Acad. Sci. USA*, **37**, 33–38.

LIVER CANCER STUDY GROUP OF JAPAN. (1990). Primary liver cancer in Japan. Clinicopathologic features and results of surgical treatment. *Ann. Surg.*, **211**, 277–287.

MCENTEE, G.P., BATTS, K.A., KATZMANN, J.A., ILSTRUP, D.M. & NAGORNEY, D.M. (1992). Relationship of nuclear DNA content to clinical and pathologic findings in patients with primary hepatic malignancy. *Surgery*, **111**, 376–379.

MARAJ, R., KEW, M.C. & HYSLOP, R.J. (1988). Resectability rate of hepatocellular carcinoma in rural southern Africans. *Br. J. Surg.*, **75**, 335–338.

MENDELSOHN, M.L. (1958). The two-wavelength method of microspectrophotometry. II. A set of tables to facilitate the calculation. *J. Cell Biol.*, **4**, 415–424.

MUNOZ, N. & BOSCH, X. (1987). Epidemiology of hepatocellular carcinoma. In *Neoplasms of the Liver*, Okuda, K. & Ishak, K.G. (eds), pp. 3–19. Springer: Tokyo.

NAGASUE, N., YAMANOI, A., TAKEMOTO, Y. & 6 others (1992). Comparison between diploid and aneuploid hepatocellular carcinomas: a flow cytometric study. *Br. J. Surg.*, **79**, 667–670.

NAKASHIMA, T., OKUDA, K., KOJIRO, M. & 4 others (1982). Pathology of hepatocellular carcinoma in Japan; 232 consecutive cases autopsied in ten years. *Cancer*, **51**, 863–877.

NAORA, H. (1955). Microspectrophotometry of cell nucleus stained by Feulgen reaction. I. Microspectrophotometric apparatus without Schwarzchild–Villiger effect. *Exp. Cell Res.*, **8**, 259–278.

OKUDA, K. & NAKASHIMA, T. (1985). Primary carcinoma of the liver. In *Bockus Gastroenterology*, 4th edn, Berk, J.E. (ed.), pp. 3315–3375. W.B. Saunders: Philadelphia.

OKUDA, K. (1990). Geographic heterogeneity of hepatocellular carcinoma. *Gastroenterol. Jpn.*, **25**, 787–792.

OKUDA, K., MUSHA, H., NAKAJIMA, Y., KUBO, Y., SHIMOKAWA, Y., NAGASAKI, Y., SAWA, Y., JINNOUCHI, S., KANEKO, T., OBATA, H., HISAMITSU, T., MOTOIKE, Y., OKAZAKI, N., KOJIRO, M., SAKAMOTO, K. & NAKASHIMA, T. (1977). Clinicopathological features of encapsulated hepatocellular carcinoma. A study of 26 cases. *Cancer*, **40**, 1240–1245.

OKUDA, K., PETERS, R.L. & SIMSON, I.W. (1984). Gross anatomic features of hepatocellular carcinoma from three disparate geographic areas. Proposal of new classification. *Cancer*, **54**, 2165–2173.

OKUDA, K., FUJIMOTO, I., HANAI, A. & URANO, Y. (1987). Changing incidence of hepatocellular carcinoma in Japan. *Cancer Res.*, **47**, 4967–4972.

PATAU, K. (1952). Absorption microphotometry of irregular shaped objects. *Chromosoma*, **10**, 341–362.

PATERSON, A.C., KEW, M.C., HERMAN, A.A.B., BECKER, P.J., HODKINSON, J. & ISAACSON, C. (1985). Liver morphology in southern African blacks with hepatocellular carcinoma: a study within the urban environment. *Hepatology*, **5**, 72–78.

SARACCI, R. & REPETTO, F. (1980). Time trends of primary liver cancer: indication of increased incidence in selected cancer registry populations. *J. Natl Cancer Inst.*, **65**, 241–247.

SCHRADER, F. & LEUCHTENBERGER, C. (1950). A cytochemical analysis of the functional interrelations of various cell structures in Arvelius albopunctatus (De Geer). *Exp. Cell Res.*, **1**, 421–452.

SUGIMACHI, K., KOGA, Y., MORI, M., HUANG, J., YANG, K. & ZHANG, R.G. (1987). Comparative data on cytophotometric DNA in malignant lesions of the esophagus in the Chinese and Japanese. *Cancer*, **59**, 1947–1950.

YOSHIDA, Y., OKAMURA, T., KANEMATSU, T., KAKIZOE, S. & SUGIMACHI, K. (1988). Comparison between microspectrophotometry and cytofluometry in measurements of nuclear DNA in human hepatocellular carcinomas. *Cancer*, **62**, 755–759.

YOSHIDA, Y., KANEMATSU, T., KORENAGA, D., SONODA, T. & SUGIMACHI, K. (1992). DNA ploidy of primary hepatocellular carcinoma and pulmonary metastases. *Clin. Exp. Metastasis*, **10**, 337–344.